# A conceptual framework to develop a patient-reported experience questionnaire on the cystic fibrosis journey in France: the ExPaParM collaborative study

**DOI:** 10.1186/s13023-023-02640-6

**Published:** 2023-02-18

**Authors:** D. Pougheon Bertrand, A. Fanchini, P. Lombrail, G. Rault, A. Chansard, N. Le Breton, C. Frenod, F. Milon, C. Heymes Royer, D. Segretain, M. Silber, S. Therouanne, J. Haesebaert, C. Llerena, P. Michel, Q. Reynaud

**Affiliations:** 1 Université Sorbonne Paris Nord (USPN), LEPS, UR 3412, 93430 Villetaneuse, France; 2 Centre de Ressources et de Compétences Mucoviscidose, Hôpital Couple-Enfants, Grenoble, France; 3 Laboratory RESHAPE U. INSERM 1290, Claude Bernard Lyon1 University, Villeurbanne, France; 4 Centre de Ressources et de Compétences Mucoviscidose, Hôpital Lyon Sud, Pierre-Bénite, France; 5 Centre de Ressources et de Compétences Mucoviscidose, CHU Lille, Lille, France; 6 Groupe Des Co-Chercheurs Patients et Parents d’enfants Atteints de Mucoviscidose, USPN, LEPS, UR 3412, 93430 Villetaneuse, France

**Keywords:** Collaborative research, Health service research, Patient experience, Patient reported experience measures, Cystic fibrosis care, Quality improvement

## Abstract

**Background:**

The objective of the study was to elaborate a conceptual framework related to the domains of patient experience along the cystic fibrosis (CF) journey from the patients and parents of children with CF to inform the design of a patient-reported experience questionnaire.

**Method:**

A collaborative research group including patients and parents with clinicians and academic researchers was set up. They identified the situations along the CF care pathway from diagnosis to paediatric care, transition to adult care and adult follow-up, transfer to transplant centres and follow-up after transplantation. Participants were recruited by CF centres in metropolitan France and overseas departments. Semi-structured interviews were conducted, transcribed verbatim and subjected to an inductive analysis conducted in duos of researchers/co-researchers using NVivo®. The conceptual framework was discussed with the research group and presented to the CF centres during two video conferences. The protocol obtained a favourable opinion from the Ethics Evaluation Committee of INSERM (IRB00003888-no. 20-700).

**Results:**

The analysis led to a conceptual framework composed of domains of the CF journey, each divided into several items. 1. CF care: Management of care by the CF centre team; in-hospital care; quality of care in the community; therapeutic education and self-management support; at-home care; new therapies and research; procreation; 2. Transplant care: management of transplant and CF care; coordination with other specialties; education and self-management support; at-home care; procreation; new therapies and research; 3. Turning points along the journey: diagnosis of CF, transition to adult care, transfer to transplantation; 4. Social life with CF: housing, employment and education, social relations, social welfare and family finances. The number of patients included and the diversity of situations made it possible to achieve a sufficient richness and saturation of codes by domain to develop patient experience questionnaires.

**Conclusion:**

This conceptual framework, resulting from the participants’ experience, will inform the design of a patient-reported experience tool, whose construct will be tested during the next phase of the ExPaParM project to assess its fidelity, intelligibility, and ability to report patient experience of the CF journey.

## Background

Cystic fibrosis (CF) is a rare genetic disease for which systematic newborn screening has been implemented in France for the past 20 years.^1^ The CF care pathway is organised by specialised paediatric CF centres and adult CF centres (in French: CRCM—"Centre de Ressources et de Compétences Mucoviscidose") within the rare disease network,^2^ and then by transplant centres when lung transplant is indicated. The French CF Registry lists patients followed in CF centres and transplant centres, records their health data, and publishes an annual data report on the evolution of their health status. According to the data from 2021, 7513 patients were registered and followed in the 45 CF centres, of whom nearly 62% were adults (4576), and 955 adults had undergone lung transplantation (representing nearly 21% of all adult patients). Between 2011 and 2018, 27 CF centres volunteered to participate in a quality improvement programme (QIP). The programme was deployed using a collaborative approach, involving patient and parent partners in collaboration with the care teams, to disseminate good care practices and to improve the health of patients, as measured by the indicators from the Registry.^3^ A research project evaluated the multiple effects of this complex intervention,^4^ including the impact of the participation of patients and parents.^5^ During a focus group in 2018, the patient and parent partners suggested that the experience of patients followed in the centres be collected extensively, in order to ensure that the difficulties encountered by socially disadvantaged groups were taken into account to guarantee better representativeness and equity. These suggestions highlighted the need for an instrument that can describe both the experience of care and life journeys and be sensitive to the improvement actions implemented.

Care pathways are commonly viewed based on the perception of healthcare professionals, and rarely from the perspective of the patients and of their families. The consequence is that this perception focuses on the 'actors' who work in patient care (CF centre medical staff, administrative and social services) and more rarely on the lived experience of patients or parents of CF children. Therefore, certain parts of the pathway are not looked into, as they are not part of what medical staff are responsible for, and fall under the patient's or the family's responsibility (at-home care, decision-making and adjustments of treatment that are part of daily life with CF). However, these factors are part of patients’ experience of the disease, and are, at least partly, the result of the care and education provided by the clinical staff. In addition, this vision, which centres on care 'actors', is of fragmented nature. Indeed, patients' experience or their level of satisfaction is usually measured when they interact with the healthcare system (at the end of a consultation, after a medical procedure or at the end of a hospital stay), when in fact the experience of patients living with a chronic illness is composed of a succession of numerous interactions with the healthcare system, which, put together, shape their experience of living with the disease. Making a diagnosis, choosing the right treatment, or arranging support in a timely manner, can only be achieved through well-organized care, and relevant and timely successive interactions with the healthcare system. Patient experience (PE), which was defined in 2014 by the Beryl Institute as "the sum of all interactions, shaped by an organization's culture, that influence patient perceptions across the continuum of care", will be analysed here across a continuum of care that centres on the patient.^6^ Approaching PE from the patient's point of view requires analysing the differences in real trajectories linked to differences in the organisation of care, and also to the environmental and socio-economic context in which patients live, as these factors are all determinants of the experience of living with the disease.^7^ When recording PE, we should look into the care pathway and its local and geographical variations, but also into the arrangements put in place in order to work, study, and find appropriate housing, as well as into the adjustments made in the family or social interactions to live with the disease. The challenge is to identify and translate this variety of life situations into the PE collection tool, in order to get information on the actual difficulties faced by patients.

The objective of the ExPaParM study is to elaborate a patient-reported experience questionnaire based on the domains and events that occur along the CF journey, which will allow future respondents to accurately trace their disease-related experience. This large-scale collection of PE will feed into the reflection of those involved in the CF pathway regarding their care and service offer, unmet needs, and the actions that could improve the experience of patients and their families.

In the first phase of the research, an exploratory qualitative study aimed to develop a conceptual framework of the domains of PE of the CF journey that are representative of a wide variety of care and life situations in France. This conceptual framework will be used in a second phase of the study to develop a patient-reported experience tool for the CF care journey. The scientific validity of the ExPaParM study is strengthened by its collaborative nature, which is based on the work of a research group that includes patients and parents of children with CF, alongside academic researchers and clinicians from CF centres who participate in therapeutic patient education programs and lead the CF network.^8^ The patients and parents who act as co-researchers are involved in association networks or communities and their personal experience covers the whole CF pathway, which serves the objective of achieving good quality research when combined with the experience of professionals from paediatric and adult centres.^9^

## Methods

The detailed protocol of the ExPaParM study has already been published.^10^ Below, we summarise the method used for the exploratory qualitative study that resulted in the elaboration of the conceptual framework of domains of patient and parent experience of the CF journey.

### Study design

#### Collaborative qualitative research

The study design is that of collaborative research, involving representatives of people living with CF, together with care professionals and academic researchers.^11^ Patients and parents involved as co-researchers are trained in the use of qualitative methods and tools to allow them to participate actively in the study. The association *Vaincre la Mucoviscidose* contributed to the recruitment of the co-researchers based on a call for application on the social networks. Five patients, including three who were transplanted at different ages, and two parents of CF children of different ages have been selected. Patient and parent co-researchers are involved in all stages of the research, from developing the protocol and interview guides to conducting semi-structured interviews, analysing verbatim transcripts, and categorising the domains of experience in N'VIVO©. They also participate in the steering committee meetings, and in various communications during conferences.

#### Description of the CF journey

**Fig. 1:**
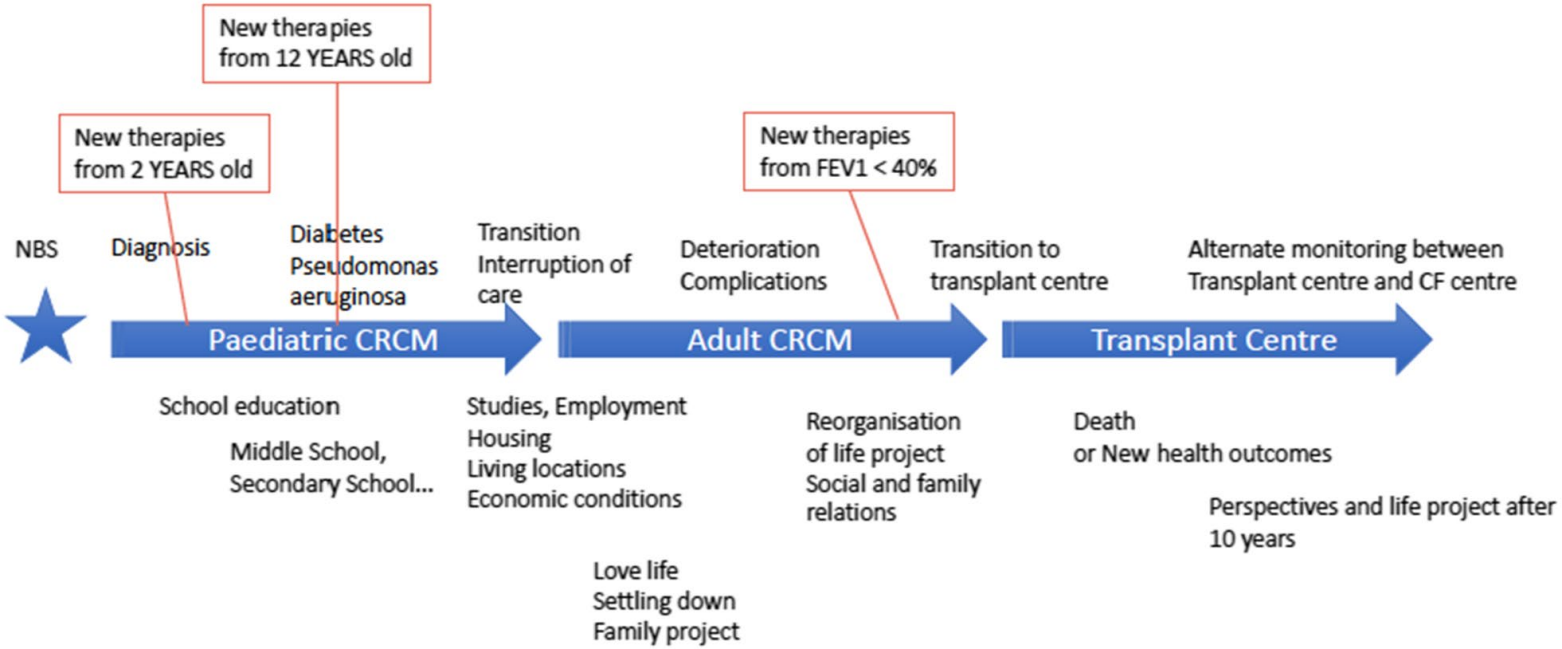
Overview of the cystic fibrosis journey from newborn screening to post-transplant care

The methodology is based on a description of the CF journey consensually developed by the research group (Fig. 1). Health events (comorbidities) are positioned according to their highest frequency of appearance in the Patient Registry data. Four groups are identified in order to prepare interview guides on appropriate topics: parents of children with CF from birth (median age at diagnosis: 1.1 months) up to 15 years of age; adolescents between 16 and 18 years of age followed in a paediatric CF centre; non-transplanted adult patients followed in an adult CF centre and transplanted adult patients followed in a transplant centre for post-transplant care. Participants should also be representative of the different situations of living with the disease. The inclusion criteria are thus defined in order to include this variety of situations in the study sample through “profiles” (Table 1) and additional criteria (Table 2) regarding patients' demographic and socio-economic conditions. The number of participants is estimated at 56, using purposive sampling, in order to represent the variety of situations and to achieve sufficient data saturation of codes and categories during the inductive analysis.

**Table 1:** List of patients’ profiles

Paediatric:	
P1 = patient between 0 and 5 years	
P2 = patient between 6 and 11 years	
P3 = patient between 12 and 15 years	
P4 = patient > 15 years and < 19 years (adolescents)	
P5 = patient who has changed CF centre in the last two years	
P6 = patient who received a CFTR modulator/potentiator	
P7 = minor patient diagnosed with COVID-19	
P8 = minor patient in the paediatric transplant process	
Adults:	
P10 = patient > 18 and < 22 years of age post- transition from paediatric care	
P11 = patient with late diagnosis in the last 4 years	
P12 = 22–26-year-old patient stabilised at CF centre	
P13 = patient with complications and progression of disease severity	
P14 = patient travelling abroad for > 3 months in the last 3 years	
P15 = patient planning to have a child or in the process of MAP	
P16 = patient with children	
P17 = patient who received a CFTR modulator/potentiator	
P18 = adult patient diagnosed with COVID-19	
P19 = patient without major complications **following transplant**	
P20 = patient with long-term complications **following transplant**

**Table 2:** Additional criteria for the patient and parent sample

**Geographic data**	
Distance between home and centre:	
0–5 km - between 5 and 20 km - between 20 and 50 km - over 50 km	
Duration of travel (hours, minutes) from home to the centre (*Hour, minutes*):	
**Family characteristics**	
Language spoken in the family of the child or adult patient:	
Marital status of the patient or parent responding to the survey:	
Number of children living in the home:	
Siblings with cystic fibrosis (*number of children*): …	
Other medical conditions in the family (*name the condition*): ………….	
**Socio-professional characteristics**	
Employment status of the patient or parent responding to the survey:	
- Salaried worker	- no professional activity
- Self-employed	- on disability benefits
- Student	- retired
Socio-professional category of the patient or parent responding to the survey:	
- Farmer or farm operator	intermediate profession
- Craftsman or trader	- employee
- Executive or intellectual profession	- worker
- Student	
Education level of the patient or parent responding to the survey:	
- No diploma	- undergraduate degree
- French certificate of general education	- master's degree
- French vocational qualifications (CAP-BEP)	- PhD level
- French general baccalaureate or professional baccalaureate	
**Specific criteria**	
- Activities with associations	- other:

**Table 3:** Number of patients involved in the study per centre

Paediatric Centre Investigator	# Patients interviewed	# Patients 0–5 y.o	# Patients / 6–11 y.o	# Patients 12–15 y.o	# Patients 16–19 y.o
		P1	P2	P3	P4
Lille Paediatric	3	1	1	1	–
Paris R. Debré	5	–	3	–	2
Strasbourg Paediatric	5	2	–	2	1
Saint-Pierre LR	5	1	2	1	1
Rennes Paediatric	6	2	1	2	1
Bordeaux Paediatric	3	1	1	1	–
Grenoble Paediatric	4	1	2	1	–
S/TOTAL Paediatric	31	8	10	8	5

Adult Centre Investigator	# Patients interviewed	# CF Patients 18–22 y.o	# CF Patients / 22 + y.o	# Transplant Patients	Of whom # Patients with child
		P10	P11-P12-13	P19-20	P15-16
Lille Adult	12	2	7	3	3
Nantes Adult	10	2	3	5	4
Lyon Adult	6	1	3	2	3
Clermont-Ferrand	6	2	3	1	3
Other CF centres	2	–	–	2	2
S/TOTAL Adults	36	8	15	13	15
TOTAL	67				

#### Study setting

CF centres have been selected based on a general agreement among the research group who considered that their patient population could allow the recruitment of patients who fit in the different criteria that were defined, and that the centre directors would accept to participate in the study. Seven pediatric centers (Bordeaux, Grenoble, Lille, Lyon, Rennes, Saint-Pierre La Réunion, Strasbourg) and 4 adult centers (Clermont-Ferrand, Lille, Lyon, Nantes) were involved in the study to recruit participants according to the profiles and criteria above:

- In rural regions (Clermont-Ferrand or east of France or southwest), patients may live far from their center (more than 2-h drive) and may not have healthcare resources locally
- In the Alps (Grenoble, Lyon) some families have to drive through the mountains to access their CF center which might be a problem in the winter
- In Paris region as well as in the north (Lille), the centers follow deprived families, a few of them being immigrants from northern Africa
- In La Réunion island, many families have specific beliefs related to genetic diseases, nutritional regimen in addition to a range of specific bacteria due to the local climate

#### Recruiting the study population

Each physician in each center proposes an anonymous list of eligible patients by profile, to protect the confidentiality of the potential participants (according to GDPR). Upon receipt of all the lists, the scientific coordinator proceeds to a harmonisation in order to ensure the representation of the various profiles. Recruitment of the patients is conducted by the partner centres, following the list adjusted by the coordinator. The individuals who are proposed but not selected on the national list are kept as potential participants who may be included later on to replace a patient failing to be contacted or to enrich the description of the categories in the course of the analysis of the data. Once a participant is included by a centre, their contact details are transmitted to the scientific coordinator to schedule the interview. The participants are asked to take part in a semi-directive interview lasting about 1.5 h. Their consent to participate in the study is obtained at the beginning of the recording of the interview. No compensation is planned for study participants.

### Collection and analysis of patient experience data

#### Semi-structured interviews

The semi-structured interviews^12^ cover the domains of care, health, and life with the disease. The experience of care and life with CF is explored in the last 18 months in order to reflect current practices in care and social domains. Transitions (diagnosis of CF, transition to adult care, transfer to transplantation) is explored in the last three years. The interview guides are available on demand. The interviews are led either by researchers or patient or parent co-researchers once trained.

#### Data analysis

The interviews are transcribed verbatims and analysed following the grounded theory approach,^13^ using N'Vivo® in an iterative manner, according to a coding framework that emerges during the process of analysis, and differently for each participant group: parents, adolescents, non-transplanted adult patients, and transplanted adult patients. Coding was done by the researchers in collaboration with patient and parent co-researchers. The coding framework established in NVivo® is articulated in categories, called 'domains', grouping 'sub-domains' of care and life with CF. As this study builds on a wide variety of care and life situations, the sample of participants is not representative of the frequency of occurrence of these situations in the total population of CF patients followed in France but allows to build the most complete conceptual framework possible. As a result, the themes that arose from analysing the interviews are not weighted in terms of their occurrence, as doing so would not reflect the importance of the themes in the total CF population.

The conceptual framework of PE domains is represented in a mind map format to be discussed in meetings with partner centres in order to gather the teams' opinion on the domains, sub-domains, and challenges faced by patients and parents.

### Ethics approval

***IRB Agreement*** no IRB00003888-Notice no 20-700

***Issue date:*** 9 June 2020

## Results

### Sample characteristics

Out of the participants included by the centres, sixty-seven participants consented to be interviewed between September 2020 and April 2021: 26 parents of children up to 15 years of age, 5 adolescents between 16 and 19 years of age, 23 non-transplanted adult patients, and 13 transplanted adult patients (Table 3). Based on the qualitative analysis of the interviews of certain profiles, and in order to enrich the description of the codes and categories, eleven additional patients were included compared with the 56 patients expected in the protocol.

### Interviews conducted with the study population sample

The 67 interviews were conducted between October 2020 and April 2021 by the researchers and some patient or parent co-researchers over the phone, due to the COVID-19 pandemic and the geographical spread of patients, representing over 80 h of audio recording (Table 4). The reality of PE during the COVID-19 pandemic was captured without overshadowing the conditions of care and life before the pandemic. The analysis of the COVID-19 experience will be the subject of a separate publication.

**Table 4:** Average duration of interviews

	Total	Number	Average	Mini	Maxi
Paediatric	39:34:44	31	1:16:36	42 min	2h07'
Adult	44:37:00	36	1:12:21	27 min	2h02'

### Mapping of PE domains and sub-domains

**Fig. 2:**
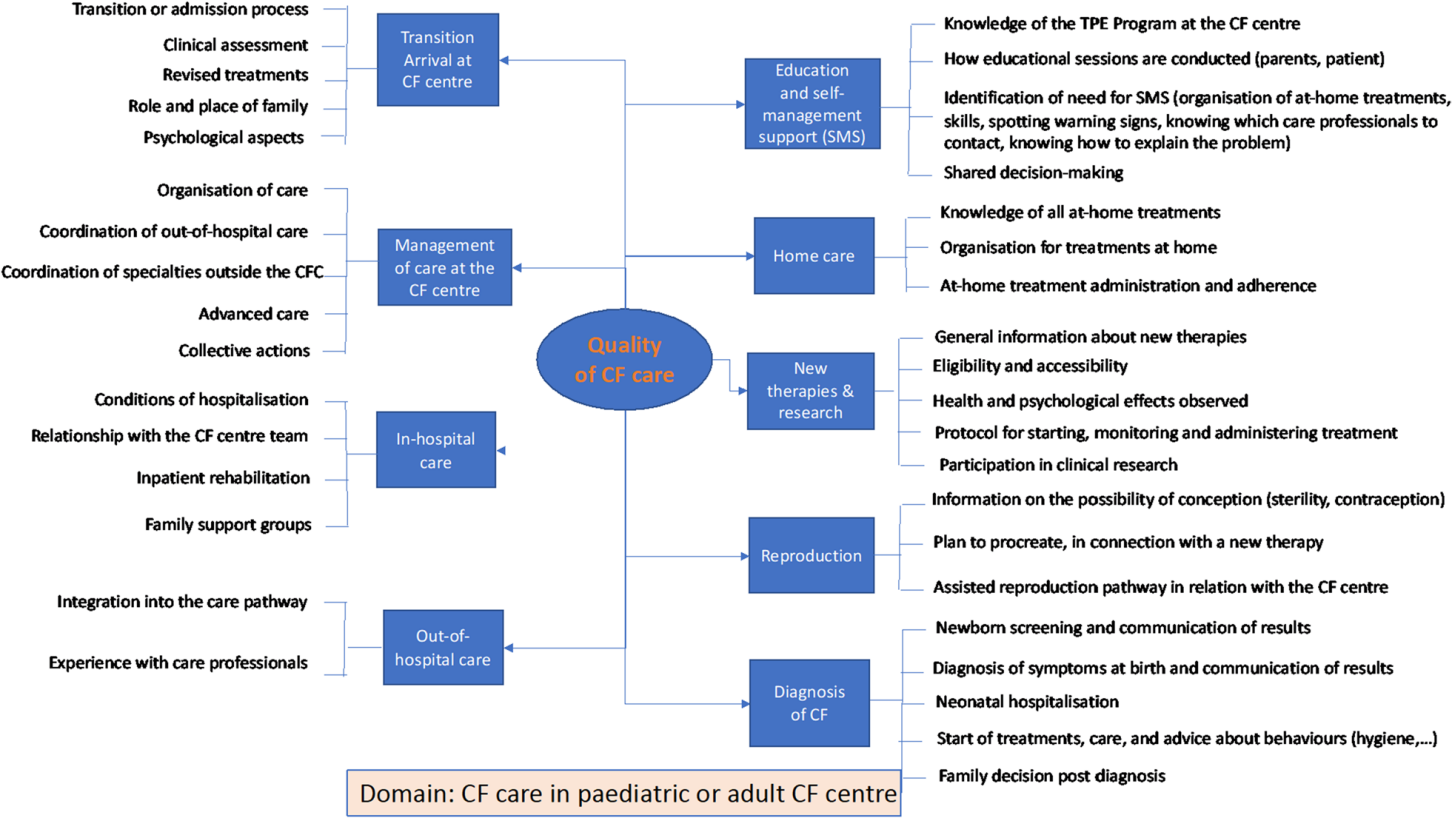
Mind map of the patient experience domains and sub-domains of the paediatric and adult CF care pathway

**Fig. 3:**
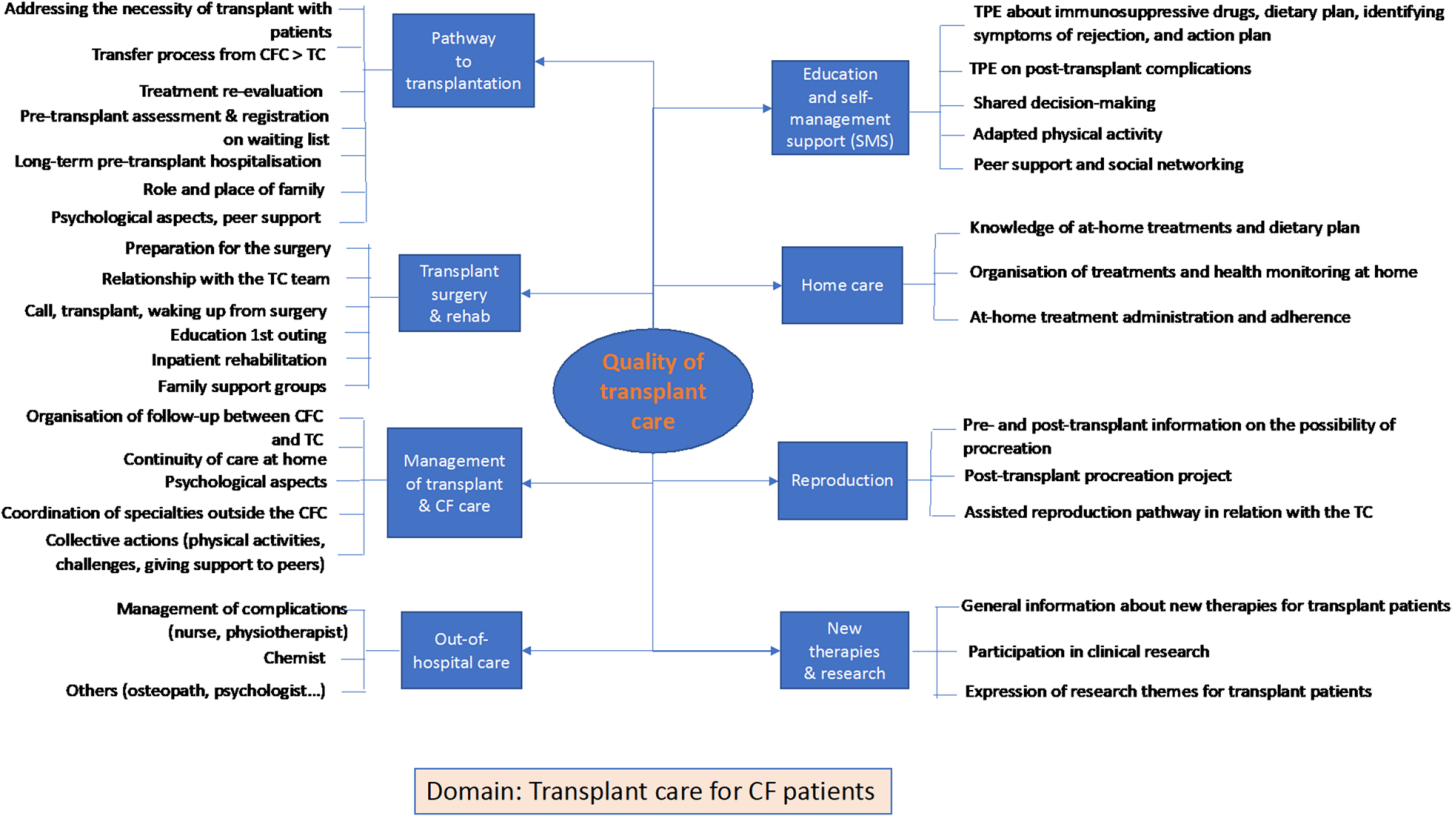
Mind map of patient experience domains and sub-domains of the adult transplant care pathway

**Fig. 4:**
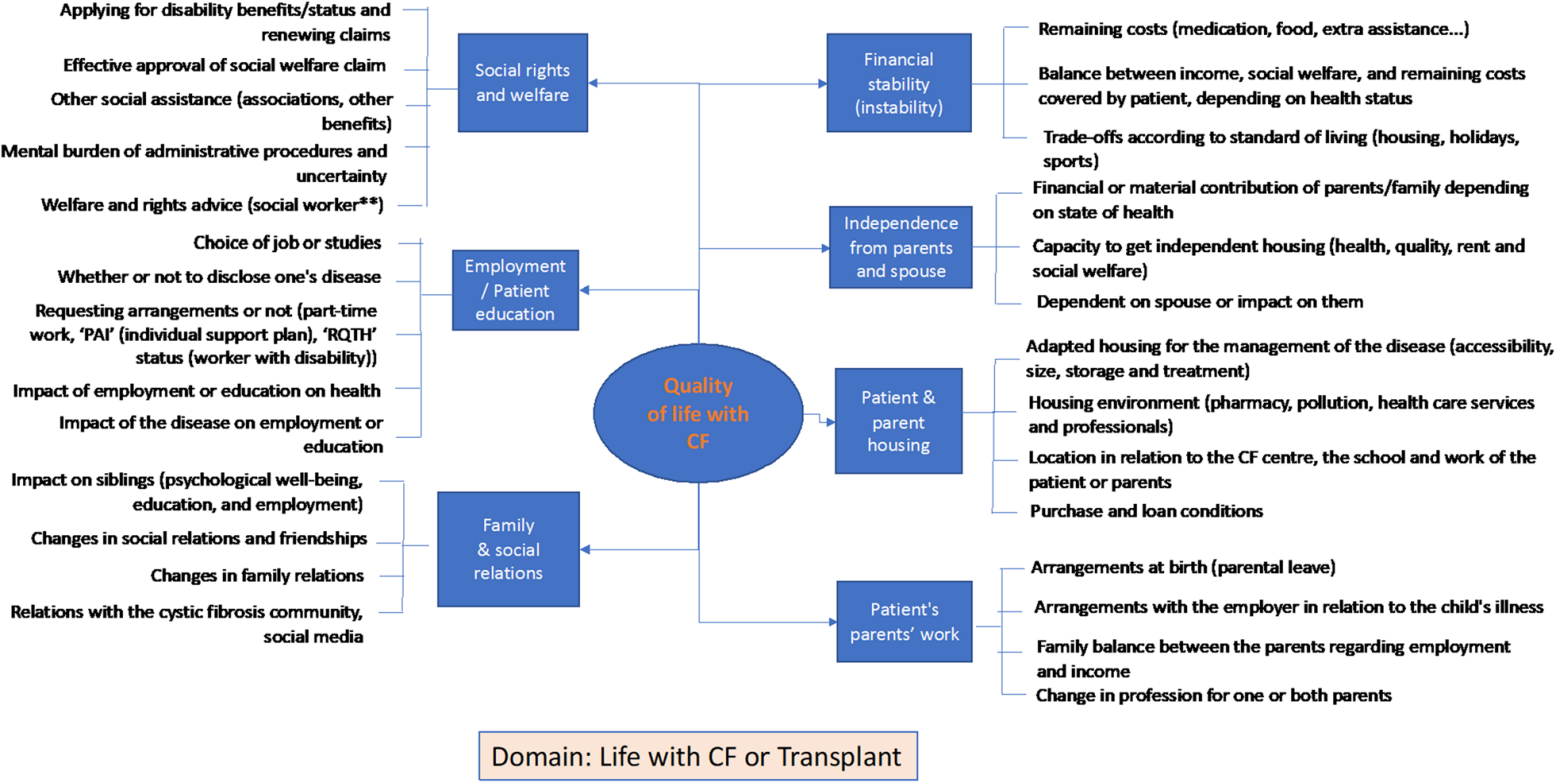
Mind map of patient experience domains and sub-domains of life with cystic fibrosis

The domains and sub-domains of the PE were summarised in three mind maps: the first one corresponds to the care pathway for paediatric and non-transplanted adult patients (Fig. 2), the second one corresponds to the care pathway for transplanted patients, starting from their transfer to a transplant centre (Fig. 3) and the third one corresponds to the domains and sub-domains of living with the disease for all patients (Fig. 4).

Presenting the paediatric and adult care pathways on the same mind map shows the common structure and steps along those pathways, but the content of the experiences depends on whether it relates to children or adults. For example, some sub-domains apply almost only to adults (Advanced care). The domain "Diagnosis of CF" only applies to parents of children under the age of 3, and "Transition to adult care" applies only to young adults who have been followed in an adult centre for less than 3 years (in accordance with the criteria). The domain of "Reproduction" includes both parents after a diagnosis of cystic fibrosis (whether they are planning to have another child with or without the use of medically assisted procreation (MAP) and adult patients (male or female) who generally undergo MAP treatment due to infertility. The content of the sub-domains also varies depending on the organisation of care and the management of comorbidities. Two domains focus on the experience of at-home care and refer to patients' management of treatments, symptoms, health deteriorations and the related decisions.

The mind map on transplant care reflects how pathways are disrupted when transitioning from a CF centre to a transplant centre (TC) once transplantation has been indicated. In general, the transfer pathway to transplantation and the burden of surgery, from its preparation to post-operative or post-rehabilitation discharge, contrast with patients' improved state of health and the lighter care required at home after transplantation. In our sample, the organisation of post-transplant follow-up is significantly different depending on whether it is centralised by TCs or it alternates between a TC and the patient's nearest CF centre. Cystic fibrosis is still present, especially in its gastrointestinal manifestations, which leads to consultations with specialists outside the transplant centre. Education in self-management and in identifying the signs of graft rejection is a central domain of patient-reported experience. Experiences of deterioration due to chronic graft rejection or the use of immunosuppressive treatments (renal transplantation, cancers) are described in the various sub-domains associated with post-transplant follow-up. Some female transplant patients can now consider childbearing, even though it remains rare. The "quality of transplant care" mind map shows that lung transplantation is not a cure, but the beginning of a new complex follow-up process with a high level of uncertainty.

The mind map on life with the disease and social aspects shows the consequences of the disease on life, but also the impact of patients' living conditions on health. The financial situation of patients or their family is closely linked to the socio-economic rights and social support. A delicate economic balance has to be struck, to allow some degree of independence of the patients from their parents or spouse. Education and employment conditions have an impact on the disease, but the reverse is also reported by respondents, as the options available to patients are often limited, depending on the environments in which they live.^14^ The home of the patient, or their family, at different stages of their life, is invested by the disease through the time spent on home treatments, adequate hygiene conditions, the level of air pollution, and the possibility of practising adapted physical activities, the proximity to employment or studies and to care professionals, and the costs associated with housing. Social and family relations are affected by the disease beyond the financial aspects, and various dynamics are observed in the closeness or distancing with members of their family or friends, depending on the perception of hygiene-related health risks, the management of their treatments or their dietary plan. Changes in family dynamics due to CF carrier screening are also reported. The "life with CF" mind map for children, adults, and transplanted adult patients overlaps with specificities of patient experience in different domains.

### Descriptive results of two domains and their sub-domains

Each domain was described with its properties (what it is composed of: sub-domains) and its various possible forms and dimensions.^15^ These descriptive results were obtained for each domain of the three mind maps: they cannot all be presented in this paper but are included in the technical report of the research. They will be used to elaborate questions for the PREM questionnaire, which will cover the events that are relevant to participants in each domain and sub-domain of the CF journey, what their experience was like, and the difficulties they faced.^6^ We selected two pivotal domains that are common to all patient profiles interviewed. The following descriptions of the two domains 'Management of CF care' and 'Therapeutic Education and Shared Decision-Making' illustrate the sub-domains and various dimensions that were reported by interviewees, as well as the differences between paediatric and adult patients.

#### Management of CF care

This domain is made up of several sub-domains: the organisation of regular visits to the CF centre; the continuity of care provided by the CF centre between visits; the annual health assessment done at the CF centre; follow-up by other specialists who are not available at the CF centre, but in relation to cystic fibrosis; the complex and advanced care associated with the deterioration of health, prior to being referred for transplant; being part of a patient or parent support group at the CF centre; the experience of moving to a different CF centre.

The sub-domain 'Management of regular visits to the CF centre' refers, on the one hand, to the personal organisation required to come to the CF centre (time and duration of the visit, managing absences from school or work, mode of transport and duration of journey from home to the CF centre), and on the other hand, the organisation of the consultation at the CF centre.

The organisation of the trip (usually every three months) is done in advance but causes difficulties with the school or employer. Consultation times can be adjusted to accommodate patients’ travel preferences. Consultations usually last two to three hours, except when delays occur, or when other consultations are added upon the arrival. For some patients, a trip to the CF centre and back also takes approximately two to three hours.

Follow-up at the CF centre continues over many years, the routine is well established. The patients' or parents' expectations regarding visits depend on (1) the state of health and the results of the examinations carried out on the same day, in particular the weight (especially for children) and spirometry, but also on (2) dietary difficulties (children), social welfare (application to be renewed), psychological or specific difficulties, and on (3) a particular event that requires the setting up of a specific care and treatment plan (holidays or trip abroad, school trip, starting the new school year…). Many topics need to be discussed once at the CF centre and it requires meeting different members of the care team (physician, nurse, physiotherapist, dietitian, psychologist, social worker). The lived experience thus depends on the response to those expectations. First of all, it depends on whether they can meet the care professionals they wish to see that day, which is made easier by advanced planning. Second, it depends on the quality of the relationship with those professionals (feeling listened to and not judged; empathy; clear responses; getting solutions to problems), and also on the possibility of shared decision-making on some examinations, medical care or treatments. And ultimately, it also depends on the perceived usefulness of the consultation in resolving current difficulties or in proposing solutions.

My FEV was yo-yoing, the IV cure was always subject to discussion: when you don't feel sick, when it thwarts projects... The negotiation was tight. There is a big work of renunciation when you do not feel the benefit of the treatment even though there are side effects of antibiotics, allergies... Gradually, I can say that there has been a taming on both sides: following an article in PubMed, I obtained that my IV cures only last 10 days. The doctor has recognized that it is important to negotiate, to convince the patient of the benefit of the treatment.

Cross-infection control when coming to the CF centre is expressed as the fear of catching some bug in the hospital. Patients and families are aware of this risk and are vigilant about the consultation conditions. Their fear depends on the physical organisation (individual consultation booth or waiting room in front of each care professional's office). When appointments are organised in order to prevent patients with CF from coming into physical contact with each other, the risk is considered low. Patients' route inside the hospital to attend consultations and the cleanliness of the rooms (hygiene, disinfection between patients) are determining factors in the perception of the risk of cross-infection, together with the wearing of masks and the use of hydroalcoholic gel. In particular, the room where sputum is coughed up is considered to be at high risk if patients cannot witness that it has been disinfected suitably.

It's simple, there are too many of us, there is no segregation (depending on the bacteria of the patients); there is a room to spit, it is called the spittoon, all the patients pass there one behind the other, no window no ventilation between two, no disinfection between patients.\

Between two visits to the CF centre, continuity of care must be organised by the centre. Outside of the consultations at the centre, patients or parents are regularly in contact with the team for different types of requests and needs, and to renew or adjust certain treatments. For example, the results of the sputum culture test taken during the visit take approximately ten days, and conditions the start of targeted antibiotic therapy. Some practices such as "We will only call you back if the results indicate that you need a change in your treatment" are perceived negatively, as they give patients the impression that their results may get forgotten about or be communicated to them late. Patients or parents contact the CF centre via e-mails and phone calls, according to a well-established procedure (phoning a dedicated number or e-mailing the nurse coordinator or the doctor directly): the response time and perceived usefulness of the answer are determining factors in patients' experience.

Annual reviews are part of the care management organised by CF centres, including complementary systematic examinations (chest X-ray, fasting blood test, abdominal ultrasound, oral glucose tolerance test…) or additional tests according to the evolution of the patient's health. Annual reviews at the hospital are reported to be key moments in the experience of care. The ability to get all tests done in one day, rather than having to come to the hospital several times, is appreciated. Indeed, having appointments spread over several days makes things more complicated, and has a negative impact on PE. To avoid doing multiple trips, some adults prefer, when possible, to do certain tests prescribed by the CF centre (blood test, oral glucose tolerance test…) in laboratories near their home, and to bring the results to the annual review. A consultation between the patient or parents and the CF centre is then usually scheduled within a month to review all results. This waiting time can cause patients or parents to experience anxiety, particularly when they expect to receive bad news (suspicion of chronicity of pseudomonas aeruginosa infection or presence of other bacteria in the sputum, carbohydrate intolerance or diabetes…). This consultation often lasts longer than usual, as the CF physician discusses the evolution of patients’ health or complications, including changes in treatment or new recommendations (physical activity, dietary recommendations) or options (therapeutic education on topics related to the test results).

Follow-up by other specialists that are not present at the CF centre, but in relation to CF, must be coordinated by the CF centre: in paediatrics, mainly ENT follow-up at the hospital or out of the hospital, and speech-language therapy (feeding problems); for adults, mainly ENT, endocrinologist, diabetologist, osteopath (chronic pain). ENT follow-up in patients with CF requires professional expertise and many patients or parents report that it often does not provide solutions. Speech therapy follow-up is considered burdensome, because it adds regular appointments to the child's schedule, but it is seen as beneficial. With the progressive appearance of CF-related diabetes (32% of adult patients have diabetes and impaired glucose tolerance is diagnosed in 20% of adolescents from the age of 10),^16^ follow-up at the CF centre includes regular diabetes screening. Such complications bring on an additional burden of monitoring and treatment and are interpreted as a worsening of patients’ health. Choosing between oral treatment or insulin therapy is a significant step and sometimes an opportunity for a shared decision-making experience. The benefits on the decrease in pulmonary infections and on weight gain are seen as positive. This compensates the disadvantages of an extra treatment, which is, in hindsight, not considered to be too burdensome compared with all the other treatments. Consulting an osteopath (often for pain reduction) is generally decided by the patients, and done outside of the CF centres, which do not have any in their teams.

In the sample, one of the patient profiles included patients whose health had deteriorated (P13), with multiple comorbidities (diabetes, resistant bacteria, liver complications, etc.) requiring advanced care characterised by the intensification of treatments, particularly antibiotics. These recurring treatments, which become less effective over time, in turn, cause serious side effects such as allergic reactions to antibiotics. This domain of the care pathway is characterised by successive bad news communicated to the patients about their deteriorating health and additional treatments. Their care becomes very burdensome, and their autonomy is reduced, leading some patients to move back with their parents or to being hospitalised for long periods of time. When patients move back with their parents, the burden for the family can be very significant and requires that parents reorganise their lives.

I was in a relationship, but at some point I was no longer independent at all, I didn't want to put the burden on my boyfriend, I went back to my parents, so that they could take care of me.

Entry into the transplant pathway is described in the transplant patients' pathway, from the moment transplantation is indicated by the CF care team and the transfer to the TC.

Parent or adult patient groups exist in some centres, of which some of the study participants are members. The existence of these collectives is known to other families, but not always all their members, and direct contact between parents or patients was rarely mentioned. The members of these collectives, who are motivated and available to explore possible improvements in care and to help patients or parents who are facing difficulties, seem however to be contacted infrequently. They are more often in contact with the CF centre teams than with the other patients or parents. They participate in information meetings (generally annual paediatrics meetings organised by the team for parents of patients followed in the centre) and are consulted on the day's programme or asked to provide information on their role.

A few families in the sample experienced a change in CF centre due to a move to a different town. This was more common among adult patients in our sample, as they sometimes had to move to university or to start a new job, and depending on when their transition to their first adult centre occurred (before or after entering university or starting a job). The determinants of a good transition experience are: feeling welcomed and listened to by the new team; the quality of the relationship between the old and the new team, which is reflected, among other things, in the transfer of patients’ records; the fact that the new team does not appear to be smaller (absence of certain specialties), less available or less expert than the previous one. The decision by a new team to change patients' treatments is experienced positively when it is explained and understood. It is a real test for the future relationship. A family who resides in French Guiana was included in our sample: as there is no specialised centre in French Guiana or Guadeloupe, follow-up is done in a paediatric CF centre in Paris, with the child and their parents coming for two to three visits per year. The parents have to cover the additional transportation costs and the care is completed by paediatric follow-up in their hometown.

#### Domain of patient therapeutic education and shared decision-making

This domain consists of several sub-domains related to either paediatric care, adult follow-up care or both: CF centres' educational programmes; the organisation of parent/patient educational sessions; the need for self-management at home; shared decision-making.

Although therapeutic patient education (TPE) has been in place since the early 2000s in CF centres and is structured around a national group named GETHEM,^17^ which was set up to work on the development of TPE when paediatric CF centres were first created, our interviews show that parents are often unaware of the term 'therapeutic education' and do not identify the sessions they attend as TPE. In our sample, no parent could cite their paediatric centre's TPE programme. When examples of educational topics were mentioned to them (nutrition and pancreatic enzymes, importance of salt intake against dehydration in children, etc.), they were able to distinguish the educational 'moment' within a consultation. Parents do not differentiate between education and information about a new treatment, or advice given during consultations. Parents spontaneously mentioned b, to which they sometimes refer as a support group: Young parent meetings; How to prepare for starting school; High-calorie meal recipes; etc. These moments of sharing and exchange as a group, and with the CF centre teams, were reported as positive experiences.

We were able to do lots of workshops, lots of questions. We all ate lunch together. We really spent the day from morning until evening with the parents, and a physiotherapist and a dietician from the CRCM who taught us all about how it works, where the disease started, which organs it could affect, and the evolution. At first, we were a little skeptical about going there, we didn't want to... well, not to cry... but we didn't want to spend a day where we were depressed all day. But they had really organized it so that we talk about the disease in general, and we can learn things we didn't know. There were a lot of things that we didn't know at all after a year... It was really very interesting.

However, these sessions held on Saturday mornings or at the end of a weekday, separately from consultations, were often seen as a reminder of the disease, and something that makes patients or parents come back to the CF centre at times that are usually reserved for leisure. The desire to attend such sessions more than once was unclear, despite patients and parents reporting positive experiences. Group sessions between children are rare, due to risks of cross-infection. When they do take place, children have to take a bacteriological test beforehand and are excluded if they carry potentially harmful bacteria. This makes the organisation of such sessions difficult, despite them being appreciated by the parents when they do take place, and it also relies on having a strong team dynamic. Concerning children’s education, parents mentioned group sessions dedicated to entering primary school or to going on field trips. Such sessions were organised with the parents and children as two separate groups, and hygiene precautions were respected. Preparing for the transition to an adult CF centre is one of the steps in the paediatric TPE programme. In our sample, adolescents had little experience of it as they were younger than 17 years of age (except for 2 individuals), the age at which paediatric transition TPE begins. The two patients over 17 years of age with severe clinical situations started Kaftrio© in 2020, with an early access authorisation. Their transition to adult CF centres was delayed in order to continue an observational study at their paediatric CF centre. However, this transition was looked into by adults who joined their adult CF centre within the last 3 years. They did not report facing any difficulties on the day of their actual transfer:

On the day we changed centre, the paediatrician accompanied us, we were introduced to the whole adult care team and there was quite a friendly atmosphere. The transition went quite smoothly. We were able to talk with our old team and the new team... It happened at an age when change doesn't cause any trauma, it was not a problem. We were pretty well informed beforehand, and I was free to transition to the new centre whenever I felt ready. At the age of 18, I was happy to change centres.

When the paediatric and adult teams meet up in order to welcome patients and their parents together, this is reassuring for them:

The doctor who used to follow me came, then my new doctor introduced himself, they were able to talk. It was interesting. And afterwards, the whole team introduced themselves. An office was assigned to me on the day, and they all came in one by one. My parents came to the new centre on the first day, to see what it was like and meet the new team, but since then, they have not felt the need to come back and I have not either.

The transfer to adult care brings along changes in follow-up, which are not always well received:

Now that I have transferred, I am more critical of the care I receive in the adult care service, which is not at all the same as in paediatrics services. For example, in paediatrics, I saw the physiotherapist every time I came to the centre. Now, this is no longer the case. I used to see many different care professionals in the paediatrics services, and I miss it at the adult care centre. Every time I come, I only see the pulmonologist and I do pulmonary function tests.

Adults did not report receiving TPE sessions after their admission to the adult centre, but they did receive information on new treatments, genetics and reproductive matters, or on certain procedures, such as the insertion of a port-a-cath for example. They did not express the need for a systematic TPE programme, but rather for reminders on self-treatments and at-home care, and reminders or clarifications on how to take certain routine medications whose effectiveness and usefulness they question. Consultations with the dietitian are identified as educational, but generally considered to be repetitive as not providing new information.

A need that is expressed in interviews was named (by the researchers) as the need for 'support for self-management at home'. On the one hand, this is expressed as a wish to make the care team realise the burden of patients' care and treatment, view things from the patients' perspective and reflect on how they would manage such a burden if they had to in their own daily lives. This underlines the complexity of the day-to-day organisation of treatments for patients, with the main challenge being to achieve optimal adherence.

It's more the aerosols that are heavy, for my hair or skin concerns, I admit that I leave them aside a little, because having done everything else, in the evening I don't want to go to do the treatment, to pass the ointment on my head... There I let go a little, I don't necessarily do it all the time, I told the dermatologist. The accumulation of everything sometimes... I prioritize care a little.

On the other hand, patients also need to be able to recognise signs that their health is deteriorating, to interpret them, and to act appropriately. They must manage such signs themselves and, if possible, learn lessons to better care of themselves next time. Some patients made notes of episodes of deterioration in a notebook to try to identify a response that seems to work for them, which they sometimes reviewed in consultation with the CF centre physician. Some seek advice from their physiotherapist, paediatrician or local GP. Some call the CF centre team or leave them a message, depending on what was agreed on beforehand. Actions are taken, but episodes are seldom analysed with the CF care teams afterwards and learning seems arbitrary. This experience was described as difficult and stressful for parents. In the case of adult patients, being both the person experiencing the symptoms, and the one who must analyse them and make the appropriate decision, makes the situation difficult. Some symptoms, such as haemoptysis, are experienced as distressing. Staying clear-headed and finding the resources to react appropriately requires anticipation and learning from experience, two elements that should be included in TPE on self-management. When the situation requires contacting the CF centre team, it is also important to describe the symptoms accurately and to assess their severity and evolution in the last few hours, during the call or in the message sent, and to anticipate the questions the professional will ask, in order to get confirmation of the course of action to adopt / or other instructions. In such circumstances, the parents or patients sometimes do not feel confident that they can explain the symptoms accurately, and they experience the situation differently depending on whether or not they feel understood, taken seriously, and that a relationship of trust is created, although remotely, to solve the problem.

And since all my blood sugar spikes were related to an infection, I wondered if it was COVID. But when I asked my doctor, he didn't want to do an additional blood test. I had to ask a doctor friend for a prescription, to see if there were antibodies... So I had to search on my own: I looked a lot on the internet to find out what are the reasons that increase blood sugar for normal diabetes and what can be the specific reasons for CF. And it's true that I was lucky, the most likely hypothesis was stress, but it could have been something else.

Self-management support also includes learning to perform certain technical procedures at home, such as airway clearance physiotherapy, or care related to enteral tube feeding. The responses received show that patients and parents consider themselves, to varying degrees, capable of performing such procedures, and feel they have, or sometimes have not, received the necessary "training" to do so. Performing technical procedures is either experienced as empowering (it allows them to travel) or as an excessive or undue burden. In our sample, parents' active participation in the organisation of care, and in care giving, makes them feel that they are part of the care team, but that their role is not always recognised by the CF centre or seems to be taken for granted. Instead, they would like to create a partnership with the care team that would result in a better recognition of their knowledge and skills, shared decision-making, and specific training.

Tom, his FEV is mediocre at 60%. But fortunately the doctor has confidence, she believes us when we tell her that when he rides a bike or a trampoline he is not out of breath... When I have a problem, I send an email or I call, they will try to answer us during the day, at worst the next day. If they don't have the answer right away, we'll get a little email: the doctor isn't there, we're trying to contact her.

Several themes were identified in paediatric or adult care, for which shared decision-making could be put in place: the decision to use intravenous antibiotic treatment (IV treatment) and its practical organisation (at the hospital/at home, schedules); when treatments are frequent, the decision to place a catheter, or insert a port-a-cath, or a PICC Line, which are invasive procedures that are sometimes reported as bad experiences, from surgical complications to reports of adverse events related to care; the decision to begin supplemental feeding and choosing between nasogastric or gastrostomy feeding; the decision to start oral medications or insulin therapy. Improved shared decisions-making would be beneficial on matters of treatments, their organisation or duration, as those conversations sometimes seem to lead to difficult or impossible negotiations with the CF centre care team. In paediatric care, disagreements regarding certain systematic protocols of IV treatments were expressed, following discussions on social media within a group of parents who compared practices between different CF centres.

I have the impression that all the centres, I don't know if they all work like that, they draw the IV treatment quite quickly I have the impression. It always went well, and we saw the improvement afterwards. But for the parents, for the child, it's always complicated to say: come on, we're going to be on IV cure for 15 days. Now, we know what it is, we were a little scared the first time...

### Main points from the patient experience collected from our study sample

We present below the main points based on positive and negative experiences identified through the analysis of the interviews. These points will have to be confirmed and, above all, weighted after the patient experience questionnaires have been administered to a representative sample of all patients followed in CF centres.

#### Main points of the care pathways

The main points of the care pathways summarised below come from a cross-sectional reading of the PE narratives by the researchers and co-researchers. They relate to the domains on the mind maps and are described in detail in the research report:

- The therapeutic partnership that patients and parents seek to build with the CF centre team contrasts with their lack of confidence or mistrust in the specialists they consult, outside the field of CF care.
- The fight against CF is a daily struggle. At home, it relies on therapeutic patient education that focuses on preventing and supporting the management of warning signs of serious complications and crisis situations. Continuity of care between two visits to the centre relies on matters of responsiveness, on the care partnership between patients or parents and the care team, and ultimately, on the stability or deterioration of patients' health and quality of life.
- The notable differences between paediatric and adult care concern: the multidisciplinary nature of paediatric care teams compared with adult care teams, which revolve around the pulmonologist—nurse coordinator collaboration; the place of the physiotherapist in adult care varies greatly between centres as well as the coordination of specialties outside the CF centre.
- The perceived risk of cross-infection during hospital visits, depending on the physical organisation of consultations, and the level of respect of infection control measures in the hospital.
- No significant experiences were reported by patients regarding disagreements between CF care team members about diagnosis or treatment.
- Difficulties were reported during inpatient hospitalisation, sometimes serious, including declarations of adverse events. These difficulties are resolved by visits from CF centre consultants at patients' bedside.
- Information on and access to new therapies varied between centres during phases of temporary compassionate use, which raises questions about access to information in patient records to verify eligibility.
- Post-transplant follow-up is organised in different ways. It is either done at the transplant centre (TC), or alternates between the TC and CF centre nearest to the patient. These differences are discussed by the patients on social media, and do not seem to be based on scientific guidelines.
- Transplant patients do not benefit from new therapies for CF and the lack of research involving this population contrasts with the fact that transplant patients are neither cured of CF, nor always in good health, and many factors make their future uncertain.

#### Main points of life with cystic fibrosis

The patients and parents in our sample have little support in resolving the social difficulties they face as if professional teams would concentrate their attention on the clinical care pathways when patients were primarily concerned by their whole journey. Hospital social workers offer little assistance in applying for welfare rights and benefits, despite some adults having difficulties renewing their social rights when they come to an end or when moving home to a new region, or little assistance in ensuring that the benefits are actually paid to them. The relationship between occupational medicine and CF centres in making workplace accommodations was not mentioned, neither regarding parents, nor adult patients who work. Some patients or families who are best informed or closest to CF patient associations do contact such groups for support. Paediatric CF centre teams usually support parents in putting in place a "PAI" (individual support plan) at their children's school. The disease creates uncertainty for the future in terms of health, but also financially for the families and patients. These uncertainties and the patient's specific needs lead to choices, made individually or as a couple, about employment, education, housing, which in turn, have a financial and psychological impact.

#### Some difficult points in transitions

The introduction of generalised neonatal screening in 2002 in France is a breakthrough that reduced the number of patients diagnosed based on symptoms in childhood or adolescence at CF centres,^18^ as being diagnosed in adulthood happens in particular situations of milder forms detected during fertility treatments. In 2021, almost all children followed in paediatric CF centres were diagnosed following newborn screening using the Guthrie test, performed within a few hours of babies' birth, and confirmed by a sweat test at about six weeks of age. In our study sample, the guidelines for announcing the diagnosis of CF and the timeframe for it are met, except in exceptional circumstances.

I would like to emphasize the kindness, the support which was great. We were completely in tears the first time we went to the hospital; the next week we were in "we're going to fight, they're going to fight with us" mode. See all this team that was dedicated to this disease. We are very lucky to have such good care.

However, young parents' experience could be improved by shortening the timeframe to get this diagnosis, in order to avoid the four to six week waiting period during which infants begin to show signs of suffering and deterioration in nutritional status, and to spare parents a lot of stress. Speeding up the process is mentioned both in terms of screening, with parents wishing to get the Guthrie test results before being discharged from the maternity, and in building a closer collaboration between the neonatal care unit and CF centres for the early diagnosis and management of CF during the hospitalisation of the newborn.

Tom was born, he was not a big baby…The only thing that stuck out to me was that when I gave him a kiss, I said to the nurse: he has salty skin. She said don't worry, we haven't given him his first bath yet, that's got to be it. That's the only thing she said to me. Afterwards, when they came to give her the Guthrie test, I asked why it was done, she explained to me and said: it takes about a month if there is something, if in a month you haven't received anything, everything is fine. On November 19 I said to my husband: everything is fine, we haven't had any mail. And on November 21, I got the call from the doctor.

The need to provide young parents with comprehensive infection prevention and control measures upon arrival at the CF centre is questioned. Indeed, providing them with information progressively over time, and in a targeted manner, according to their environment and child's age and degree of mobility, could prevent young parents from making radical and harsh decisions that may influence the psychological well-being of the family.

We were advised to change the sponge too, while doing the dishes. No more classic yellow sponge with green, it is better to use dishcloths or things like that that dry quickly. We were told to avoid plants in the house too, to avoid standing water in cups or things like that. In the car, on the way back from the CF centre, we decided to get rid of our pets and our plants. We were also told to disinfect all the siphons once a week, to prevent bacteria from forming in the siphons and taps. At first we were like, wow, that's a whole lot more to pay attention to. And finally, once you get used to it, it happens naturally.

Especially since we observe that such measures have an impact on family dynamics, which can lead to young parents being isolated and can cause increased psychological difficulties.

“When you go to other people's house, it won't necessarily be as clean as your house, it's not too good to put them in a bubble either.”

Young adults who experienced a transition in the last few years did not indicate noticing a change in their care between their admission at the adult CF centre and the following years. A gradual evolution in their care could help them become autonomous progressively, particularly in the organisation of their treatments at home. The change in CF centre is often connected to a move due to university studies, and away from the parents' home. Young adults report organising all of their care near their new home, while studying or working. This stage in life and adult care is reported to be a time of risk for medication non-adherence or even lapses in care, which are directly related to a deterioration in patients' health.

The transfer pathway to transplantation, as well as the post-transplant follow-up, are sometimes organised differently. Indeed, the information provided to patients and the tests that are part of the assessments required by CF centres and TCs may vary. As there are fewer TCs that take on patients with CF than adult CF centres, the referral process follows rules that seem unclear to patients and their families, and which they assume are based on geographical criteria or pre-transplant comorbidities (bacteria). Patients get information from their peers, from the CF centre or on social media. They noticed that staff in some CF centres have little knowledge of the transplant pathway and little connexion with the TC teams, which is sometimes combined with a feeling of failure towards the care they received at the CF centre.

When I was transplanted, I came back to my CF centre, we both talked a lot with the doctor, and that's when she told me that the announcements of transplants, she is really very bored to do them. She said to me: “you can't imagine how difficult it is to announce to your patients that you are trying to save their lives day by day and all of a sudden to tell them that you can't do anything anymore, and they have to go to a transplant center, and we don't know if the transplant will work. Maybe so, but there can be complications. I couldn't tell you everything. I couldn't tell you there are some who died.

Patients' loss of autonomy in the phase prior to transplantation forces them to move closer to their families for support, or even to move back into their parents' home. Despite the preparation done with the TC team, the circumstances in which patients will get the call for transplant, as the surgery cannot be scheduled in advance, remain a source of worries, and some expenses have to be paid for by the patient (travel costs). Reducing waiting times on the transplant list make it all the more crucial to prepare patients early, including psychologically, so that they are not unprepared when they get the call for transplant.^19^

## Discussion

Our exploratory qualitative study, which used a diverse sample of CF patients or parents of children with CF between October 2020 and April 2021, enabled the elaboration of a conceptual model of patient experience of the CF pathway. Given the time period studied (last 18 months for patient follow-up and last 3 years for transitions), the study shows a cross-sectional view of PE of the current care pathway. This study also reports on people's experience of various events along their life with the disease. The conceptual model and the descriptions of domains and sub-domains will enable the elaboration of questionnaires tailored to each audience (parents, adult patients and transplanted adult patients) in order to collect the experience of numerous patients and parents in the future, with the aim of providing sufficient insight for the care teams to reflect on the improvements needed in the fields of care and support.

The collaborative nature of the research allowed for a wide variety of health and life situations to be represented, allowing the recruitment of patients (or their parents) aged between 6 weeks to over 50 years, thus covering all stages of the disease. This variety of situations was complemented by taking into account socio-economic criteria and by selecting centres that follow socially disadvantaged or culturally diverse populations. For example, our sample reflects the care pathway between French Guiana and metropolitan France, several transplantation pathways between Réunion Island and metropolitan France, and the trajectories of families who came from North Africa or the Middle East to have their children treated. The participants also represent diverse socio-economic classifications and levels of income. The fact that interviews took place over the phone did not hinder the process of collecting the sample’s experiences, even during the COVID-19 pandemic. The experiences gathered highlighted events, positive aspects, and difficulties faced along the pathway, from our sample. While the patient and parent co-researchers were not representative of such diversity, beyond that of representing different stages of the care pathway, recruiting the study sample according to the inclusion criteria ensured that the data collected was representative of diversity. The challenge of representing diversity lies in the ability to elaborate a patient experience questionnaire that reflects the entire population concerned by CF in France.^20^ Based on the conceptual framework of domains and sub-domains and their content, which was derived from the experiences that emerged from interviews, the questions formulated are intended to collect, as a survey, the experience of numerous patients and parents. This way, the frequency of situations and difficulties faced will be understood on a larger scale and discrepancies may appear between centres or groups of patients, depending on socio-economic criteria or therapeutic approaches (benefiting from new therapies such as Kaftrio© or not).

The patients and parents in our sample have strong knowledge of the disease and treatments and learn continually about their effects. They have the necessary skills to cope with stressful situations and have a strong understanding of the risks and of infection control measures, which all come from experience. Such knowledge and skills, which develop progressively along their care and life pathways, and through their relationship with CF centre care teams, lend credibility to the information they reported during interviews, and to their perception of events they experience. The questions and hypotheses formulated by them on the causes of symptoms, the risks associated to certain behaviours or situations, and the effects of treatments, are part of their ongoing reflection. When presented with the findings and mind maps, the entire research group and partner CF centres did not identify any inconsistencies or discrepancies, based on their own expertise, that would call into question the findings from the interviews. On the contrary, these observations were an opportunity to confirm the difficulties faced by some of their patients, of which the care teams were aware, the difficulties in accessing certain resources at the CF centres, and the limits of what care professionals can do in the domains outside of CF care.

Analysing interviews with the co-researchers of the research group helped to identify difficulties faced in singular experiences, with a specific focus on aspects of life at home, intra-family relations, transitions and certain care pathways, and of life with the disease. Some co-researchers expressed the wish that the future questionnaires be used by the association or by the CF centre patient groups in their dealings with the centres, or with health authorities, in order to support their advocacy work to improve care pathways. Therefore, the future questionnaire would constitute an inventory of the expected standards of care to which patients are entitled. It would question the 'actors' of care, not only on their actions with patients, but also on the consequences of these actions in patients' lives, the patients' capacity to self-manage, the sharing of information between healthcare professionals and how it is coordinated along patients' pathways, and the support provided to patients to cope with health difficulties and with the necessary adjustments to be made in their lives. Consequently, the results collected will reflect the complexity of the healthcare system, thus leading to systemic reflections at the meso and macro levels of healthcare. The responses to be provided should not be limited to one actor or even one territory but must be articulated between the different levels of the healthcare system in order to avoid perpetuating inequalities across the territory, even if actions are implemented locally. How can actions be prioritised in light of the PE results collected? The following criteria can be used to prioritise certain objectives: the frequency of the problems reported by patients, the seriousness of the consequences on patients' health, and patients' safety. Disparities in PE results between territories or healthcare facilities or patient groups (population segmentation analyses) may be a priority remedial action to identify potential best practices and restore equity of access to higher quality care. In addition, in order to prioritise objectives, actions must be aligned at the different levels of the system, for example, between a financing mode, availability of qualified resources, and a consultation for addressing and implementing a complex treatment.

The study method led to the exploration of slices of patients' lives with CF. The juxtaposition of events of daily life allows us to reconstruct patient experience of their entire trajectory. However, the questionnaire that will be developed will have to be administered differently according to respondents’ profile (and not in its entirety) by targeting the stages of the care pathway that they have gone through in the last 18 months, and the transitions experienced in the recent past. Thereby, the questionnaire will be tailored to each patient, based on their previous answers to certain 'filter' questions.

### Study limitations

Although this exploratory qualitative study included a large number of participants, our sample remains limited and cannot cover all situations of care and life with CF in France. However, the conceptual framework developed reflects the entire pathway, with the exception of the adolescent pathway between the ages of 17 and 18 that is characterised by the preparation for transition to an adult CF centre. In order to develop this part of the questionnaire, we will rely on recent publications on CF^21^ or rare diseases^22^ to include suitable questions.

## Conclusion

To our knowledge, our study is the first one to explore the entire CF care pathway from the perspective of patients' and parents' experience. This approach could be applicable to other rare diseases in order to describe and analyse the pathways from the point of view of patients and their families. The richness of the patient experience collected and analysed shows that it is difficult to reduce it to a generic PREM questionnaire. Indeed, it encompasses domains and sub-domains outside of healthcare institutions and questions life with the disease as a continuous process. In addition, the numerous and various events that occur along the pathway make it necessary to collect precise data, in order to work with healthcare teams on the difficulties of managing the disease and their consequences in patients' lives. Comparing the French CF questionnaire with international tools will enable us to compare the domains and sub-domains identified in order to highlight similarities or specific features.^23^,^24^

## Organisational structure and responsibilities

PROMOTER – IN PARTNERSHIP WITH – CF CENTRES

Laboratory of Health Practices and Educations UR3412, Sorbonne Paris Nord University, France.

Laboratory RESHAPE, INSERM, Claude Bernard Lyon 1 University, France.

1-CRCM Pédiatrique de Grenoble—Dr Catherine LLERENA.

2-CRCM Pédiatrique de Paris Robert Debré—Dr Michèle GERARDIN.

3-CRCM Pédiatrique de Lille—Dr Nathalie WIZLA.

4-CRCM Pédiatrique de Rennes—Dr Eric DENEUVILLE.

5-CRCM Pédiatrique de Bordeaux—Dr Stéphanie BUI.

6-CRCM Pédiatrique de Strasbourg—Dr Laurence WEISS.

7-CRCM Pédiatrique de Saint Pierre La Réunion—Dr Caroline PERISSON.

8-CRCM Adulte de Lyon – Dr Quitterie REYNAUD.

9-CRCM Adulte de Clermont-Ferrand – Dr Isabelle PETIT.

10-CRCM Adulte de Lille – Dr Olivier LE ROUZIC & Dr Anne PREVOTAT.

11-CRCM Adulte de Nantes—Dr Isabelle DANNER.

## Footnotes

1 https://www.ameli.fr/assure/sante/themes/mucoviscidose/diagnostic-evolution

2 Définition formelle de création des CRCM issue de la circulaire no 502 du 22 octobre 2001.

3 Pougheon Bertrand et al. [1].

4 Pougheon Bertrand et al. [2]

5 Pougheon Bertrand et al. [3]

6 Wolf et al. [4].

7 Being a patient. First report of the Patients Association’s patient experience programme. The patients association, July. 2020. https://www.patients-association.org.uk/Handlers/Download.ashx?IDMF=16708179-90d6-41dd-a360-2c53b7e9ebe7

8 Jagosh et al. [5]

9 Bergold, Jarg & Thomas, Stefan [6]

10 Pougheon Bertrand et al. [7]

11 Glaser BG, Strauss AL [8]

12 Legard et al. [9]

13 Birks M, Mills J [10].

14 Fernagu Oudet, [11]

15 Paillé [12].

16 https://www.vaincrelamuco.org/sites/default/files/registre_francais_de_la_mucoviscidose_bilan_2021.pdf

17 https://educationtherapeutique.muco-cftr.fr

18 Rosenfeld M, Ostrenga J, Cromwell EA, et al.[13]

19 K.J. Ramos, P.J. Smith, E.F. McKone, et al. [14]

20 Osagie Ebekozien, [15]

21 V. Vion Genovese, M. Perceval, L. Buscarlet-Jardine et al., [16]

22 Les 5 dimensions de la transition dans le champ des maladies rares. Filières de santé maladies rares (2020) [17]. 4 pages. https://drive.google.com/file/d/1wDfVWMSg5cl7OHeENdR9fZyGzh7MImEh/view

23 J.J. Noordhoek, J. Jeyaratnam, D. Zomer et al., [18] Development and outcomes of a patient driven cystic fibrosis quality of care improvement project, Journal of Cystic Fibrosis, https://doi.org/10.1016/j.jcf.2022.07.004

24 Homa et al. 19.]

## Declarations

### Access to the study data

The study data is stored in a secured environment at Sorbonne Paris University under NVivo® licence. The database received registration (n°98) in the computer processing directory of the University on Tuesday, 16th March 2021.
